# Estimands in published protocols of randomised trials: urgent improvement needed

**DOI:** 10.1186/s13063-021-05644-4

**Published:** 2021-10-09

**Authors:** Brennan C. Kahan, Tim P. Morris, Ian R. White, James Carpenter, Suzie Cro

**Affiliations:** 1 MRC Clinical Trials Unit at UCL, London, UK; 2 Imperial Clinical Trials Unit, Imperial College London, London, UK

**Keywords:** Estimand, Randomised controlled trial, Statistical analysis, Intercurrent events, Truncation-by-death, Protocol

## Abstract

**Background:**

An estimand is a precise description of the treatment effect to be estimated from a trial (the question) and is distinct from the methods of statistical analysis (how the question is to be answered). The potential use of estimands to improve trial research and reporting has been underpinned by the recent publication of the ICH E9(R1) Addendum on the use of estimands in clinical trials in 2019. We set out to assess how well estimands are described in published trial protocols.

**Methods:**

We reviewed 50 trial protocols published in October 2020 in *Trials* and *BMJ Open*. For each protocol, we determined whether the estimand for the primary outcome was explicitly stated, not stated but inferable (i.e. could be constructed from the information given), or not inferable.

**Results:**

None of the 50 trials explicitly described the estimand for the primary outcome, and in 74% of trials, it was impossible to infer the estimand from the information included in the protocol. The population attribute of the estimand could not be inferred in 36% of trials, the treatment condition attribute in 20%, the population-level summary measure in 34%, and the handling of intercurrent events in 60% (the strategy for handling non-adherence was not inferable in 32% of protocols, and the strategy for handling mortality was not inferable in 80% of the protocols for which it was applicable). Conversely, the outcome attribute was stated for all trials. In 28% of trials, three or more of the five estimand attributes could not be inferred.

**Conclusions:**

The description of estimands in published trial protocols is poor, and in most trials, it is impossible to understand exactly what treatment effect is being estimated. Given the utility of estimands to improve clinical research and reporting, this urgently needs to change.

**Supplementary Information:**

The online version contains supplementary material available at 10.1186/s13063-021-05644-4.

## Background

A recent trial evaluating the use of cabazitaxel in patients with metastatic prostate cancer found it significantly improved quality of life as assessed by the EQ-5D (estimated mean difference 0.08) [1]. However, readers might be surprised to learn this effect does not represent what they might expect, namely the anticipated benefit of cabazitaxel if introduced as part of usual care. Instead, it is an estimate of what the treatment effect is expected to be in the hypothetical setting where men with metastatic prostate cancer never experience disease progression or death. This is due to a quirk of the study methods: first, investigators stopped collecting quality of life data after patients experienced disease progression, and so data after progression was set to missing (as was data for patients who had died), and secondly, they then analysed the available data using a repeated-measures mixed-model, which implicitly imputed what the missing data would have been had patients been alive and not progressed [2].

While it is debatable whether there is value in knowing the effect of a new cancer treatment in the hypothetical setting where patients never die nor experience disease progression (the very thing most treatments aim to prevent), it is clear that understanding exactly what treatment effect is being estimated is essential to the proper interpretation of study results. But while statistical methods used for analysis are required for the protocol and trial publication [3–6], a careful statement of the exact question being addressed by each analysis is not, and it is therefore left to readers to try and piece together what is being estimated.

The cabazitaxel trial above is merely an illustrative example used to highlight a widespread issue that is not unique to this particular study. This lack of clarity is particularly problematic, as even with expert statistical knowledge, it can be challenging to understand what treatment effect is being estimated (Table 1). Many factors can change the interpretation of estimated treatment effects in subtle ways, including the trial design [7–9], how the data are analysed [10–13], and how issues such as treatment discontinuation or treatment switching are handled [14–16]. A reader might reasonably expect that if a protocol indicates the analysis population includes all randomised participants, then the resulting treatment effect should equally apply to all participants. However, this is not necessarily the case; some statistical methods will include all randomised participants in the analysis, but the treatment effect will only apply to a subset of participants (e.g. complier-average causal effects are undertaken on the full trial population, but apply only to the subset of participants who would comply under either treatment strategy [17]). The converse can also be true, where some patients may be excluded from the analysis, and yet the estimated treatment effect still applies to the entire trial population under certain assumptions [18].

**Table 1:** Examples of challenges in determining the estimand based on the method of analysis in several fictional trials

Summary of analysis method	Key parts of estimand
Time to non-fatal myocardial infarction will be analysed using a Cox regression model. Patients who die before experiencing an event will be censored at the time of death.	Hazard ratio in the hypothetical setting where patients do not die. This is because the Cox model assumes that censored patients are still alive and at risk of non-fatal myocardial infarction.
Quality of life (using EQ-5D) will be collected at the beginning of every chemotherapy cycle, until disease progression or death. Quality of life will be analysed using a repeated-measures mixed-model, with treatment, time point, and their interaction included as fixed effects, and patient as a random effect. Intention-to-treat analysis (all available data analysed according to allocated treatment group) will be used.	Difference in the means in hypothetical setting where patients do not die and do not experience disease progression. This is because data is set to missing after disease progression or death, but the repeated-measures mixed-model implicitly imputes what the missing data would have been had patients been alive and not progressed.
Difference in the mean blood pressure at 6 months will be analysed using a complier-average causal effect (CACE) analysis. All randomised patients will be included in the analysis.	Difference in the means in the subset of patients who would have complied under both treatment conditions. This is because while complier-average causal effect analysis is undertaken on the full trial population, it only applies to the subset of participants who would comply under either treatment strategy.

In response to these issues, the International Council for Harmonisation released a guidance document on the use of estimands in randomised controlled trials (RCTs) and other studies (e.g. single-arm trials and observational studies) as an addendum to ICH E9 (*ICH E9(R1) Addendum on estimands and sensitivity analysis in clinical trials to the guideline on statistical principles for clinical trials*) [14]. An *estimand* is a precise description of the treatment effect to be estimated (i.e. it can be thought of as the precise question that the analysis method is trying to answer). It involves specification of five attributes: (i) population of interest, (ii) treatment conditions to be compared, (iii) outcome measure of interest, (iv) population-level summary measure denoting how outcomes under different treatment conditions will be compared (e.g. by a risk ratio, odds ratio, or difference in proportions), and (v) how intercurrent events not addressed in aspects (i)–(iii), which may affect the interpretation or the existence of outcome data, such as treatment discontinuation, use of rescue medication, or death, will be handled. Importantly, these five attributes are defined in relation to the treatment effect we *want* to estimate and are distinct from how these attributes may be handled in the actual statistical analysis (for instance, our population of interest for the estimand may be all trial participants, even though our statistical analysis may exclude participants with missing data [18]).

Separating the statistical analysis (the method of answering the question) from the question itself (the estimand) has several benefits. It can (i) clarify which treatment effect is being addressed by each trial analysis, which may otherwise be opaque to readers; (ii) ensure that chosen treatment effects are relevant to patients and other stakeholders; and (iii) ensure the design, conduct, and analysis of the trial are aligned with the research objectives [19–27]. The aims of this paper are to evaluate how well estimands are described in a set of published RCT protocols and to determine whether it is possible to infer the exact treatment effect being estimated for each trial’s primary outcome.

## Methods

The ICH E9(R1) Addendum (which states the primary estimand should be documented in the trial protocol) was published in draft form in 2017 and as a final version in November 2019 [14]. We reviewed the 50 most recently published protocols in *Trials* and *BMJ Open* on the date of our search (October 20, 2020), in order to evaluate the description of estimands in trial protocols nearly 1 year after the final version and 3 years after the draft version of the ICH E9(R1) Addendum was published. We chose *Trials* and *BMJ Open* as they publish the majority of RCT protocols.

One author (BCK) identified and assessed protocols for eligibility by hand-searching the journal websites article lists, sorted by publication date order.

Articles were eligible for inclusion if they were a full protocol of a randomised trial in humans. The exclusion criteria were (i) pilot/feasibility trial, (ii) phase I/II trial, (iii) dose-finding study, and (iv) trial in patients with COVID-19.

We excluded trials of interventions for COVID-19, as due to the urgency of the pandemic situation in 2020, we anticipated these protocols will have been developed more quickly than others, and so may be atypical and less likely to have described estimands.

No formal sample size calculation was conducted, but based on a preliminary screen of protocols outside the target date range, clear trends were observed, indicating that 50 protocols would be sufficient to identify major deficiencies in the reporting around estimands.

### Data extraction

Data from each protocol was extracted independently by two reviewers, each with more than 10 years of experience in clinical trials. Disagreements were resolved by discussion. We extracted data on how well the primary estimand for the trial’s primary outcome was described using a pre-piloted standardised data extraction form. We determined the primary outcome as follows:

- If only one outcome was listed as the primary, we used this.
- If multiple (or no) outcomes were listed as the primary, but only one outcome was used in the sample size calculation, we used this.
- If multiple (or no) outcomes were listed as the primary and no sample size calculation was performed, or a sample size calculation was performed for multiple outcomes, we used the first clinical outcome listed in the objectives/outcomes section.

Similarly, we determined the primary estimand as follows:

- If only one estimand was listed, we used this.
- If multiple (or no) estimands were listed, we used the main analysis of the primary outcome to identify the estimand. If there was not a single analysis approach identified as the primary, we used the first analysis approach listed in the statistical methods section.

### Determining whether each estimand attribute was stated/inferable/not inferable

For each trial’s primary estimand, we determined whether each of the five attributes comprising the estimand was explicitly stated in the protocol, not explicitly stated but inferable, or not inferable.

We judged an attribute as explicitly stated if it was clearly described as part of the estimand or as part of the objective of the trial. For instance, if the protocol included a description of the estimand which stated that a difference in the means would be used for the population-level summary measure, this attribute would be classified as explicitly stated. Similarly, if the protocol did not include a formal description of the estimand, but stated “the objective of this trial is to determine whether the intervention increases the mean difference in the quality of life compared to the control”, then the population-level summary measure attribute would also be classified as explicitly stated.

We judged attributes as inferable if they were not explicitly stated as part of the estimand or trial objectives, but could be inferred based on the statistical methods section or other sections of the protocol (i.e. we could construct an estimand based on what was written). For instance, if the protocol indicated an intention-to-treat approach would be used for analysis (all patients included in the analysis, analysed according to their allocated treatment arms), we would infer the population attribute of the estimand as pertaining to all patients who met the inclusion/exclusion criteria (unless this was combined with an analytical approach, such as complier-average causal effects, which we knew to target a different population), the treatment condition(s) aspect as the treatments as assigned regardless of any deviations, and that a treatment policy strategy pertained to the handling of all non-truncating intercurrent events (e.g. treatment discontinuation and use of rescue medication, but not mortality) provided outcome data collection continued post-intercurrent event (i.e. the protocol explicitly stated this, or stated outcome data would be collected for all participants and did not indicate data collection would stop after any intercurrent event). Similarly, if the statistical methods section stated they would estimate an odds ratio, or that data would be analysed using a logistic regression model (and this information had not been included as part of the trial’s objective), we inferred the population-level summary measure as an odds ratio.

We judged attributes as not inferable if it was not clear to us how to reconstruct the estimand based on what was written in the protocol. For instance, if the statistical methods section did not state which patients or data points were to be included in the analysis, we classified the population attribute as not inferable. Similarly, if the protocol stated that patients with treatment deviations were to be excluded from the analysis, we set the handling of intercurrent event attribute to be not inferable, as it was not clear to us whether this corresponded to a hypothetical strategy (the treatment effect in the hypothetical situation where the intercurrent event did not occur) or principal stratum strategy (the treatment effect in the subset of participants for whom the intercurrent event would not occur). In this latter case, we also set “population” to not inferable, as we could not determine whether the entire population under a hypothetical strategy was of interest, or whether a principal stratum population was of interest; however, we set “treatment” to inferable, as the specific treatment condition attribute could be inferred based on the exclusions. Finally, for the population-level summary measure, we required a measure of magnitude (e.g. difference in means, hazard ratio, risk difference); if the statistical methods section stated the outcome was to be analysed using a statistical test only (e.g. Fisher’s exact test or a log rank test), we set the population-level summary measure to not inferable.

We classified intercurrent events as (i) non-adherence (e.g. treatment discontinuation, missed doses, or not starting treatment), (ii) treatment switching (e.g. switching from placebo to the active intervention), (iii) mortality (where the outcome of interest no longer exists after the point of death), and (iv) other intercurrent events (e.g. rescue medication, changes to treatment which are not classified as non-adherence, or use of non-trial treatments).

Handling of non-adherence was extracted for all trials. For the other types of intercurrent events (treatment switching, mortality, and other), we first determined whether these were applicable to the trial; if so, we then extracted data on them. Mortality was judged as applicable if it was listed as an outcome (or component of an outcome), or any section of the protocol indicated that some patients may die. Treatment switching and other intercurrent events were assumed to be not applicable (i.e. not expected in the trial) unless they were mentioned as expected anywhere in the protocol.

When the handling of intercurrent events was not stated, we checked to see whether we could infer which of the five strategies listed in the ICH E9(R1) Addendum the analysis method corresponded to treatment policy (where the occurrence of intercurrent events is considered irrelevant), hypothetical (e.g. the scenario where the intercurrent event does not occur is envisaged), composite (the intercurrent event is part of the outcome definition), while-on-treatment (the outcome prior to the intercurrent event is used), or principal stratum (e.g. the population in whom the intercurrent event status would be the same, regardless of treatment group, is of interest).

We collected data on all relevant intercurrent events for each trial; however, as per the ICH-E9(R1) Addendum, we considered only those intercurrent events not explicitly stated in either the treatment condition/population/outcome attributes in order to evaluate the “intercurrent events” attribute. For instance, if a protocol explicitly stated its treatment condition attribute as “the intervention as assigned, regardless of non-adherence”, we evaluated the intercurrent events attribute based on the other relevant intercurrent events besides non-adherence. However, if relevant intercurrent events were inferable (but not stated) as part of the treatment condition/population/endpoint attributes, we also included these as part of the “intercurrent events” attribute.

We defined the overall estimand as either stated (if all five attributes were stated), inferable (if all five attributes were either inferable or stated, but not all were stated), or not inferable (if at least one of the five attributes was not inferable).

## Results

We performed the search on 20 October 2020. We assessed 73 protocols in total; 10 were ineligible because they were pilot/feasibility trials, 6 were phase I/II trials, one was a dose-finding study, and 6 were trials in patients with COVID-19. The 50 included protocols were published between 5 and 19 October 2020.

The characteristics of the included trials are available in Table 2. Most trials (*n* = 46/50, 92%) had an academic or not-for-profit sponsor. Results broken down by academic/not-for-profit vs. pharmaceutical sponsor are available in the supplementary appendix.

**Table 2:** Characteristics of the included trials

Question	Trials (*n* = 50)
Journal—no. (%)	
*Trials*	16 (32%)
*BMJ Open*	34 (68%)
Sponsor—no. (%)	
Pharmaceutical/for-profit	4 (8%)
Academic/not-for-profit	46 (92%)
Crossover trial—no. (%)	0 (0%)
Factorial trial—no. (%)	0 (0%)
Non-inferiority/equivalence trial—no. (%)	1 (2%)
Cluster trial—no. (%)	7 (14%)
Intervention type—no. (%)	
Pharmacologic	6 (12%)
Surgical	5 (10%)
Psychosocial/behavioural/educational	18 (36%)
Others	20 (40%)
Multiple types	1 (2%)
Planned sample size—median (IQR)	266 (120, 548)

### Description of estimands

None of the 50 protocols made any attempt to explicitly describe the estimand (Table 3), and in 74% of trials, we could not infer the estimand. In only 53% of trials were we able to infer four or more estimand attributes.

**Table 3:** Description of the primary estimand

Question	Trials (*n* = 50)—no. (%)
Used term “estimand”	0 (0%)
Cited ICH E9(R1) Addendum	0 (0%)
**Estimand attributes**	
Population	
Stated	0 (0%)
Inferable	32 (64%)
Not inferable	18 (36%)
Treatment condition(s)	
Stated	0 (0%)
Inferable	40 (80%)
Not inferable	10 (20%)
Outcome	
Stated	50 (100%)
Inferable	0 (0%)
Not inferable	0 (0%)
Population-level summary measure	
Stated	0 (0%)
Inferable	33 (66%)
Not inferable	17 (34%)
Handling of intercurrent event(s)	
Stated	0 (0%)
Inferable	20 (40%)
Not inferable	30 (60%)
**Overall estimand**	
Stated	0 (0%)
Inferable	13 (26%)
Not inferable	37 (74%)
**Number of attributes not inferable**	
0	13 (26%)
1	14 (28%)
2	9 (18%)
3	13 (26%)
4	1 (2%)
5	0 (0%)

In all 50 protocols, the outcome attribute was stated as part of the trial objectives; however, no other attribute was explicitly stated as part of the trial objectives in any protocol. In 36% of trials, we could not infer the intended population; in 20% the treatments of interest, in 34% the population-level summary measure, and in 60%, we could not infer how intercurrent events such as non-adherence would be handled.

### Reasons why attributes were or were not inferable

The results are shown in Table 4. Reasons why the population was not inferable were that the analysis population was not clearly described (*n* = 11), or that patients with treatment deviations were excluded, and we could not infer whether this was meant to correspond to the entire population (under hypothetical compliance) or to a subpopulation of compliers (*n* = 7).

**Table 4:** Reasons why attributes of estimands were inferable or non-inferable

Question	Trials—*n*/*N* (%)
***Population***	
Inferable (*N* = 32)	
Inferred as all eligible participants based on ITT description	32/32 (100%)
Not inferable (*N* = 18)	
Analysis population not clearly described	11/18 (61%)
Participants with treatment deviations excluded from analysis, but unclear whether target population was all patients (under hypothetical compliance) or subset of compliers	7/18 (39%)
***Treatment condition(s)***	
Inferable (*N* = 40)	
Inferred treatment policy based on ITT description	33/40 (83%)
Inferred intended treatment based on the exclusion of certain deviations	7/40 (18%)
Not inferable (*N* = 10)	
Unclear how treatment deviations will be handled in analysis	9/10 (90%)
Unclear which treatment strategy planned analysis corresponds to^a^	1/10 (10%)
***Population-level summary measure***	
Inferable (*N* = 33)	
Inferred from the type of regression model	17 (52%)
Stated type of summary measure they would estimate	16 (48%)
Not inferable (*N* = 17)	
Analysis strategy not clearly described	8 (47%)
Statistical test only	9 (53%)

^a^Actual dose of concomitant treatment given in each arm to be included as a covariate in the regression model; unclear what intended treatment strategy this approach corresponds to

Reasons why the treatment condition attribute was not inferable were that it was unclear how treatment deviations would be handled in the analysis (*n* = 9) or that it was unclear which treatment strategy the planned analysis approach corresponded to (in one protocol, the analysis model included the dose given of a concomitant treatment as a covariate, and we could not infer whether they were interested in the treatment at a particular dose, the treatment if intervention patients received the same dose of concomitant treatment they would receive under intervention, or in a treatment policy approach).

Reasons why the population-level summary measure attribute was not inferable were that the planned analysis strategy was not sufficiently clearly described (*n* = 8) or that only a statistical test would be used, with no corresponding treatment effect or measure of magnitude (*n* = 9).

### Description of handling of intercurrent events

The results are shown in Tables 5 and 6. Handling of non-adherence was inferable in 34 protocols (68%), in each case because the authors planned an intention-to-treat analysis and collected post-deviation outcome data, allowing us to infer a treatment policy strategy. Reasons it was not inferable were (i) that it was not clear how deviations would be handled in the analysis (*n* = 9) and (ii) that patients with deviations were to be excluded from the analysis, and we could not infer whether the authors intended this to correspond to a hypothetical strategy (e.g. the treatment effect if the deviations had not occurred) or a principal stratum strategy (e.g. the treatment effect in the subset of patients who would not deviate under either treatment) (*n* = 7).

**Table 5:** Description of handling of intercurrent events for the primary estimand

Question*	Trials—*n*/*N* (%)
Non-adherence/discontinuation	
Applicable	50/50 (100%)
Stated	0 (0%)
Inferable	34 (68%)
Not inferable	16 (32%)
Treatment switching	
Applicable	3/50 (6%)
Stated	0/3 (0%)
Inferable	3/3 (100%)
Not inferable	0/3 (0%)
Mortality	
Applicable	20/50 (40%)
Stated	0/20 (0%)
Inferable	4/20 (20%)
Not inferable	16/20 (80%)
Other intercurrent events	
Applicable	10/50 (20%)
All stated	0/10 (0%)
All inferable	7/10 (70%)
Not all inferable	3/10 (30%)

^*^Non-adherence/discontinuation is defined as applicable for all trials. Mortality was defined as applicable if it was listed as an outcome (or component of an outcome), or the protocol indicated some patients may die. Treatment switching and other intercurrent events were assumed to be not applicable (i.e. not expected in the trial) unless they were mentioned as expected in the protocol

**Table 6:** Reasons why handling of intercurrent events was inferable or non-inferable

Question	Trials—*n*/*N* (%)
***Non-adherence***	
Inferable (*n* = 34)	
Analysis by ITT, inferred as treatment policy	34/34 (100%)
Not inferable (*n* = 16)	
Unclear how treatment deviations will be handled in the analysis	9/16 (56%)
Participants with treatment deviations excluded, but unclear whether the intention is to target hypothetical or principal stratum strategy	7/16 (44%)
***Treatment switching***	
Inferable (*n* = 3)	
Analysis by ITT, inferred as treatment policy	3/3 (100%)
***Mortality***	
Inferable (*n* = 4)	
Inferred as a composite strategy	4/4 (100%)
Not inferable (*n* = 16)	
Unclear how mortality will be handled in the analysis	16/16 (100%)

Only three protocols mentioned treatment switching as a potential issue. In all three cases, we inferred a treatment policy strategy based on an intention-to-treat analysis approach.

Mortality was mentioned as a potential intercurrent event in 20 protocols (40%). In four protocols, we inferred a composite approach based on the technical definition of the outcome used in the methods section of the protocol (we listed these as inferable rather than stated because the less technical outcome definition listed in relation to the trial objectives did not include mortality). In the remaining 16 protocols, the statistical analysis section made no mention of how mortality would be handled, and we therefore could not infer a strategy.

Only 10 protocols (20%) mentioned any other intercurrent events as possibilities. In total, these protocols mentioned 15 other types of intercurrent events (Table 7). These were change to assigned treatment (*n* = 4) and use of a non-trial treatment (*n* = 10), and one trial of in vitro fertilisation mentioned a lack of embryos to transfer to the participant as a potential issue.

**Table 7:** Description of other intercurrent events

Question	
Number of trials with ≥ 1 other intercurrent event	10
Total number of other intercurrent events	15
Type of other intercurrent events	
Change to assigned treatment	4
Use of non-trial treatment	10
No embryos to transfer in an IVF trial	1
Planned strategy to handle other intercurrent events (*n* = 15)	
Stated	0/15 (0%)
Inferable	12/15 (80%)
Not inferable	3/15 (20%)
Reason inferable (*n* = 12)	
Analysis by ITT, inferred as treatment policy	12/12 (100%)
Reason not inferable (*n* = 3)	
Unclear how intercurrent events will be handled in the analysis	1/3 (33%)
Participants with intercurrent events excluded, but unclear whether the intention is to target hypothetical or principal stratum strategy	1/3 (33%)
Unclear which strategy intended analysis corresponds to^a^	1/3 (33%)

^a^Actual dose of concomitant treatment given in each arm to be included as a covariate in regression model; unclear what intended treatment strategy this approach corresponds to

The strategy to handle the other intercurrent events was inferable in 12 cases (as a treatment policy strategy based on an intention-to-treat analysis). In three cases, it was not inferable; in one case, it was unclear how the event would be handled in the analysis; in one case, participants with the intercurrent event were to be excluded from the analysis, and we could not infer whether they intended a hypothetical or principal stratum strategy; and in one case, it was unclear which strategy the planned analysis corresponded to (where the analysis model was to include the actual dose given of a concomitant treatment as a covariate, described above).

## Discussion

The use of estimands substantially clarifies the exact treatment effect being addressed by each analysis, so ensuring that both the questions being addressed from each analysis are meaningful to patients and other stakeholders, and alignment between the question (estimand) and the methods used to answer the question (the trial’s design, conduct, and analysis). However, despite the publication of the draft ICH E9 Addendum in 2017 and the final version in 2019, we found no evidence of uptake in our review of protocols published in October 2020. No protocols referenced the ICH E9(R1) Addendum, used the term “estimand”, or attempted to formally describe the estimand for their primary outcome. Further, in 74% of trials, there was not enough information to infer the estimand, leaving it unclear as to what precise treatment effect the trial was estimating.

The one attribute which was well described was the outcome, which was mentioned as part of the trial’s objectives in each of the 50 protocols. However, none of the other four attributes was explicitly described in any protocols, and there was often insufficient information to infer them. In particular, the handling of intercurrent events could not be inferred in 60% of protocols. The main reasons for this were that it was often not stated how intercurrent events would be handled in the analysis or it was simply stated that participants with intercurrent events would be excluded, but with no clarification as to whether a hypothetical or principal stratum approach was intended. In particular, the handling of mortality was very poorly described. Of the 16 trials in which mortality was a potential intercurrent event but was not part of a composite outcome, none of them described how it would be handled in the analysis. This was likely driven by the fact that a treatment policy strategy cannot be used for mortality (as the relevant outcome data no longer exist), and so handling of mortality must be explicitly stated in the text, instead of being inferred based on an intention-to-treat analysis, as can be done for other (non-truncating) intercurrent events, such as treatment discontinuation or use of rescue medication.

Interestingly, a precise description of the statistical methods to be used was not always sufficient to allow us to infer the treatment effect being estimated (or to put it another way, knowing the method of answering the question does not necessarily tell us *what* question is being answered). This was most notable when participants with treatment deviations were excluded, which could correspond to several different estimands as discussed above, but also occurred in other settings (e.g. when one trial adjusted for an actual dose of treatment received, which could also correspond to several different estimands of interest).

Furthermore, even though we as statisticians were able to infer certain attributes of the estimand based on the statistical methods section in some protocols, this does not mean that the treatment effect being estimated was clearly described. This inference often requires knowledge of the mechanics behind statistical analysis methods (e.g. that repeated-measures mixed-models implicitly impute data after death), which those relying on trial results to inform healthcare decisions (such as patients, clinicians, or those writing evidence guidelines) may not have. In fact, many statisticians may not fully understand the mechanics behind the methods they use. As such, understanding of what treatment effects are being estimated by the people using the trial results is likely to be lower than what we have found here.

Additionally, even if we are able to infer *what* is being estimated from the trial methods, this does not necessarily correspond to what investigators *wanted* to estimate. For instance, in a trial evaluating a new treatment for prevention of some non-fatal adverse event which will be analysed using a Cox model, investigators may censor patients who die before experiencing the event of interest out of convenience. However, the implication of this analytical choice is to estimate the effect of treatment in the hypothetical setting where patients do not die. Although in some cases this may be what investigators want to estimate, in many cases, it is not, and so the use of estimands can help them to assess whether their planned analysis approach corresponds to the desired treatment effect.

There are several potential explanations of why estimands are not being used. The ICH E9(R1) Addendum was a collaboration between global regulatory bodies and the pharmaceutical industry and was aimed primarily at pharmaceutical trials being used for regulatory submissions. Our review contained mostly academic trials, and there are, to our knowledge, no current guidelines aimed at academic trials that promote the use of estimands. As such, many academic trialists may simply be unaware of the concept of estimands (although it should be noted that none of the four industry-sponsored trials in our review did not report the estimand clearly). It is also likely that some of the protocols included in our review were written before the final ICH E9(R1) Addendum was published and have not been updated since. However, this does not explain the poor description of the methods which meant we could not infer the estimand in almost three quarters of protocols.

Further, Mitroiu et al. [23] reviewed documents relating to applications for drug authorisations to determine how well estimands were described and which strategies to handle intercurrent events were being used. They found that the estimand was rarely explicitly described and usually had to be inferred based on the statistical methods section and other parts of the protocol. They also found there was often a mismatch between the estimand strategies being used and those recommended by disease guidelines.

As others have described, different estimands may be required by different stakeholders [7, 14, 19, 22], and so multiple estimands may be of use. Choice of estimand should be made in collaboration between relevant stakeholders, rather than left to the statistician alone based on their preferred analytical approach.

## Conclusions

The description of estimands in published trial protocols is poor, and in most trials, it is impossible to understand exactly what treatment effect is being estimated for the primary outcome. Given the potential for estimands to enhance clarity and improve the trial design, their use should be mandated by funding bodies, journals, and within reporting guidelines such as CONSORT and SPIRIT.

## Supplementary Information

**Additional file 1: Table S1.** Description of primary estimand by sponsor type.

## Data Availability

The datasets used and/or analysed during the current study are available from the corresponding author on reasonable request.

## Abbreviations

ICH: International Council for Harmonisation
RCT: Randomised controlled trials
