# Supplementary material for: Estimands in published protocols of randomised trials: urgent improvement needed
Source: Trials. 2021 Oct 9;22:686. doi: 10.1186/s13063-021-05644-4 (PMC8500821; doi:10.1186/s13063-021-05644-4)
Supplement: Supplementary file 1 — Additional file 1: Table S1. Description of primary estimand by sponsor type. [file 13063_2021_5644_MOESM1_ESM.docx]

# **Supplementary material for:** *Estimands in published protocols of randomised trials: urgent improvement needed*

**Table S1 – Description of primary estimand by sponsor type**

| **Question** | **Academic/not-for-profit (n=46)** | **Pharmaceutical/for-profit (n=4)** |
| --- | --- | --- |
| **Estimand attributes** |  |  |
| Population |  |  |
| Stated | 0 (0) | 0 (0) |
| Inferable | 31 (67) | 1 (25) |
| Not inferable | 15 (33) | 3 (75) |
| Treatment(s) |  |  |
| Stated | 0 (0) | 0 (0) |
| Inferable | 38 (83) | 2 (50) |
| Not inferable | 8 (17) | 2 (50) |
| Outcome |  |  |
| Stated | 46 (100) | 4 (100) |
| Inferable | 0 (0) | 0 (0) |
| Not inferable | 0 (0) | 0 (0) |
| Population-level summary measure |  |  |
| Stated | 0 (0) | 0 (0) |
| Inferable | 32 (70) | 1 (25) |
| Not inferable | 14 (30) | 3 (75) |
| Handling of intercurrent event(s) |  |  |
| Stated | 0 (0) | 0 (0) |
| Inferable | 19 (41) | 1 (25) |
| Not inferable | 27 (59) | 3 (75) |
| **Overall estimand** |  |  |
| Stated | 0 (0) | 0 (0) |
| Inferable | 13 (28) | 0 (0) |
| Not inferable | 33 (72) | 4 (100) |
